# Supplementary material for: Drivers of female power in bonobos
Source: Commun Biol. 2025 Apr 24;8:550. doi: 10.1038/s42003-025-07900-8 (PMC12022330; doi:10.1038/s42003-025-07900-8)
Supplement: Supplementary file 2 — Supplemental Information [file 42003_2025_7900_MOESM2_ESM.pdf]

Supplemental material to DRIVERS OF FEMALE POWER IN BONOBOS

Community	Year	Males [>10y] (>10 =< 15y)	Females	# decided dyadic aggressions	# female coalitions	Proportion days with 1<MTF	Proportion females win	# intersexual dyads with decided aggressions	FDI DS	Female party size
Ekalakala	2016	3(0)	6	31			0.18	6		
Ekalakala*	2017	3(0)	7	248	3	0.55 (N=352)	0.36	16	0.5	5.83
Ekalakala*	2018	3(0)	8	96	13	0.68 (N=338)	0.32	18	0.33	3.84
Ekalakala*	2019	3(0)	8	438	1	0.43 (N=346)	0.48	19	0.58	5.45
Ekalakala*	2020	3(0)	8	103	10	0.47 (N=109)	0.91	10	0.71	6.49
Ekalakala	2021	3(0)	9	63			0.45	9		
Fekako*	2019	4(0)	3	487	2	0.20 (N=312)	0.7	12	0.75	2.21
Fekako*	2020	3(0)	3	253	0	0.05 (N=95)	0.67	7	0.67	2.67
Fekako	2021	3(0)	3	230			0.65	6		
Kokoalongo	2016	9(2)	13	28			0.73	9		
Kokoalongo*	2017	10(2)	14	197	24	0.73 (N=322)	0.76	47	0.85	7.55
Kokoalongo*	2018	9(2)	13	276	15	0.60 (N=330)	0.75	58	0.83	4.73
Kokoalongo*	2019	6(2)	13	592	46	0.37 (N=319)	0.86	52	0.97	6.22
Kokoalongo*	2020	4(0)	12	94	58	0.73 (N=109)	0.98	17	1	3.59
Kokoalongo	2021	4(0)	12	16			0.50	5		
Bompusa West*	2007	9(4)	11	131	2	0.34 (N=64)	0.69	23	0.76	5.07
Bompusa West*	2008	9(4)	11	454	14	0.31 (N=206)	0.65	53	0.72	4.87
Bompusa West*	2009	9(4)	11	285	7	0.26 (N=138)	0.6	37	0.71	5.39
Bompusa West	2010	4(4)	13	79			0.55	19		
Bompusa West	2013	7(1)	14	236			0.57	38		
Bompusa West	2014	7(1)	14	33			0.21	10		
Bompusa West	2015	7(1)	14	93			0.34	31		
Bompusa East*	2018	7(3)	11	237	2	0.10 (N=148)	0.43	59	0.60	3.78
Bompusa East*	2019	7(2)	11	406	4	0.16 (N=171)	0.39	57	0.57	5.34
Eyengo	1993	7(1)	16	78			0.65	13		
Eyengo	1994	6(1)	14	40			0.87	9		
Eyengo	1995	7(2)	16	47			0.67	14		
Eyengo	1996	6(1)	15	23			0.75	4		
Eyengo	1997	7(1)	11	50			0.73	20		
Eyengo	1998	6(0)	11	10			1	5		

SI Table 1. Overview of study communities, years, and number of adults. * Indicates group-years in which information on female coalition formation and sexual swellings was available for detailed analyses of the influence of coalition formation and reproductive synchrony on female power (MODEL 2/3). “Proportion days with > MTF” stands for the proportion of the yearly

observation days (number in brackets) with more than 1 maximally tumescent female in the group. Female power within groups is either measures by the *proportion of female win*, the proportion of conflicts between males and females won by females or by the *FDI DS*, a female dominance index which indicates the percentage of males in a group outranked on average by each female using hierarchies based on all decided agonistic interactions observed in the group using the David's score.

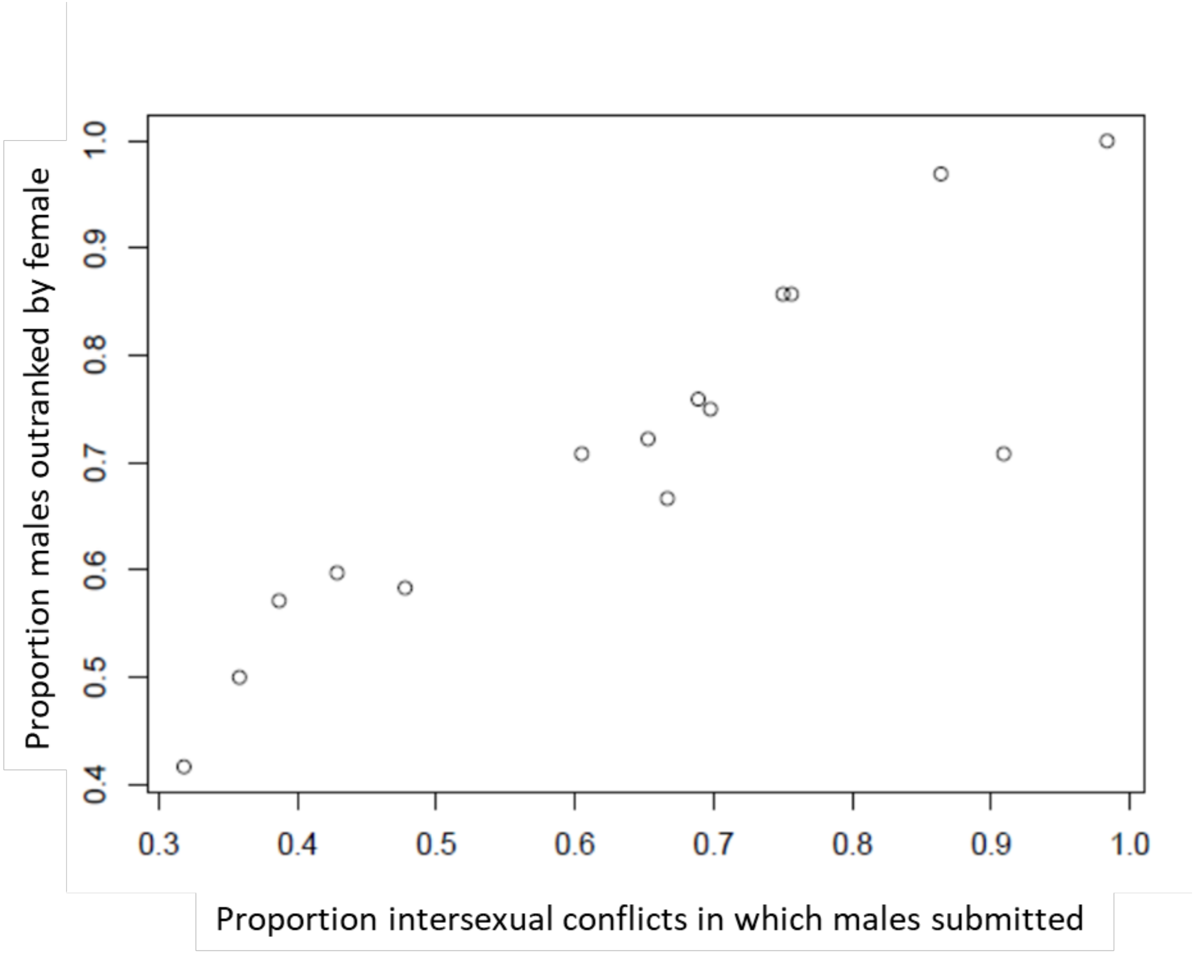

SI Figure 1. Relationship between the percentage of intersexual conflicts in which males submitted to females within a community/year and the average proportion of males in a community outranked by each female [based on David Score] (Spearman's rank correlation rho = 0.89, P<0.001).

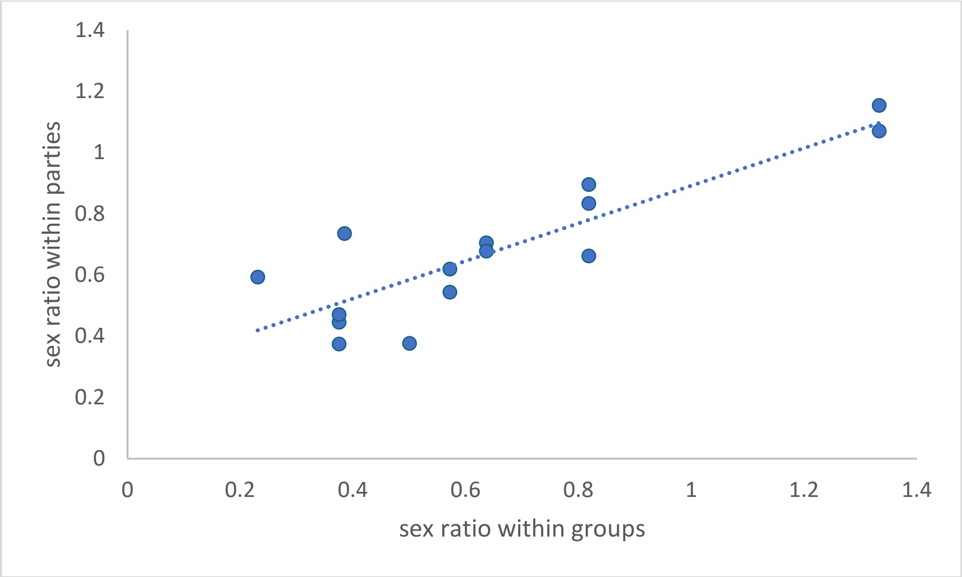

SI Figure 2. Relationship between sex ratio within groups and sex ratio within parties

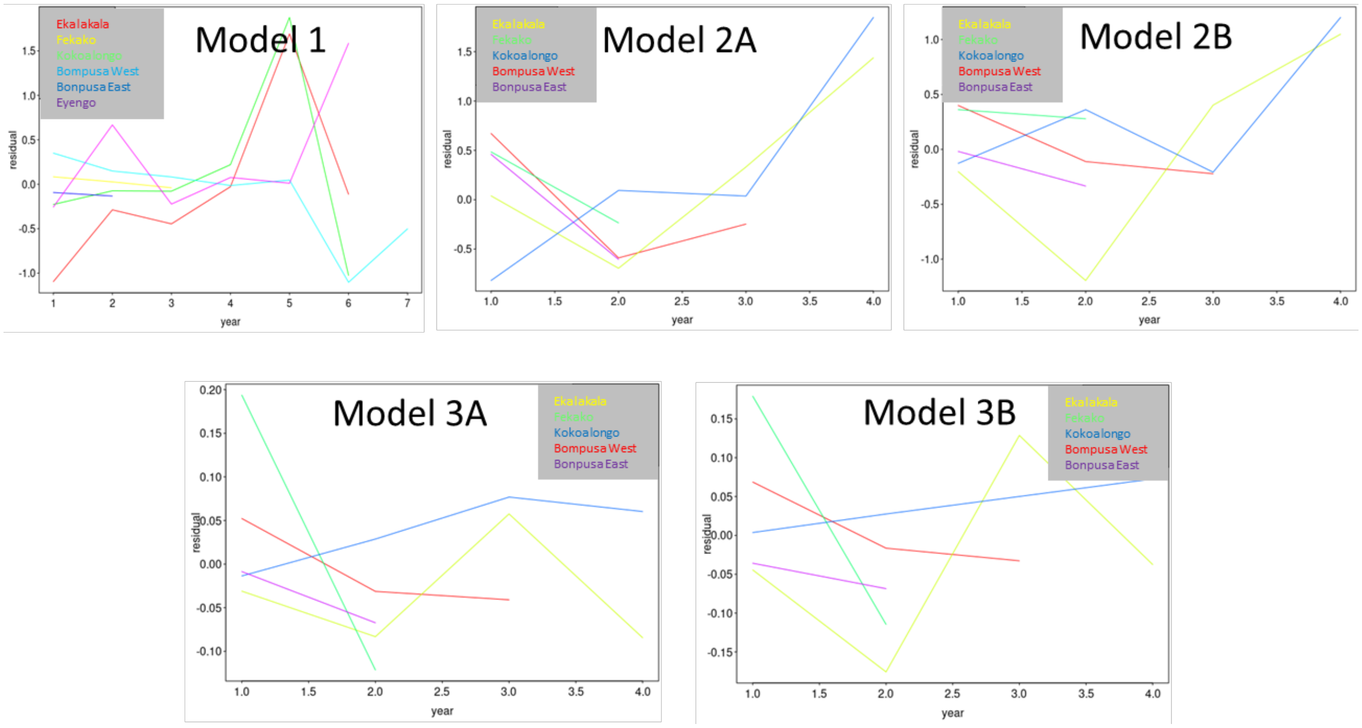

SI Figure 3. Residuals of the community term in consecutive years in the respective models.

	Model 2C			Model 3C		
	5 communities, total 15 years			5 communities, total 15 years		
Response	Intersexual conflicts in which males submitted to females within a community/year			Average percentage of males in a community outranked by each female [based on David Score]		
Full-null model	LRT, $\chi 2$ = 3.802, df = 2, P=0.149			LRT, $\chi 2$ = 6.329, df = 2, P=0.042		
	Est	SE	P	Est	SE	P
Intercept	0.523	0.181		0.889	0.248	
Female coalition frequency against males	0.739	0.307	0.016	0.332	0.145	0.022
Proportion of males	0.025	0.221	0.91	-0.09	0.259	0.726
Random factor	Community, observation level			Community, observation level		
Random slope	Female coalition frequency			Female coalition frequency		

SI Table 2. shows an overview of the structure and results of the 2 models analyzing the link between the two measures of female dominance and the propensity of females to form coalitions and the proportion of males in the communities.

A	Model 2Aff->m			Model 2Bff->m		
	5 communities, total 15 years					
Response	Intersexual conflicts in which males submitted to females within a community/year			Intersexual conflicts in which males submitted to females within a community/year		
Full-null model	LRT, $\chi2$ = 16.395, df = 2, P<0.001			LRT, $\chi2$ = 3.170, df = 2, P = 0.205		
	Est	SE	P	Est	SE	P
Intercept	0.777	0.204		0.633	0.27	
Female coalition frequency against males	0.784	0.152	<0.001	0.819	0.209	<0.001
Percentage days with 2 or more maximally tumescent females	0.056	0.358	0.875	- 0.113	0.225	0.615
Random factor	Community, observation level			Communities, observation level		
Random slope	Percentage days with 2 or more maximally tumescent females			Female coalition frequency		

B	Model 3Aff->m			Model 3Bff->m		
	5 communities, total 15 years					
Response	Average percentage of males in a community outranked by each female [based on David Score]			Average percentage of males in a community outranked by each female [based on David Score]		
Full-null model	LRT, $\chi2$ =9.483, df = 2, P = 0.009			LRT, $\chi2$ =4.753, df = 2, P = 0.092		
	Est	SE	P	Est	SE	P
Intercept	0.97	0.286		0.858	0.256	
Female coalition frequency against males	0.491	0.124	0.001	0.449	0.171	0.009
Percentage days with 2 or more maximally tumescent females	-0.131	0.24	0.584	- 0.233	0.199	0.24
Random factor	Community, observation level			Community, observation level		
Random slope	Percentage days with 2 or more maximally tumescent females			Female coalition frequency		

SI Table 3 A/B shows an overview of the structure and results of the 4 models analyzing the link between the two measures of the degree of female power over males (Models 2: Intersexual conflicts in which males submitted to females within a community/year; Models 3: Average percentage of males in a community outranked by each female [based on David Score]) and the predictor variables of the propensity of females to form coalitions against males and the

synchrony of maximally tumescent females. Models A and B differ in the structure of the random slope term.

CODE FORMAL ANALYSIS

```
library(lme4)
library(car)
library(kyotil)
library(glmmTMB)
xdata=read.csv(*SI Table 1)
xx=lmer(female_DS_dom~freq_ff+perc_m2_ef+(1+perc_m2_ef||group), data=xdata,
REML=F)
xx=powerTransform(xdata$freq_ff, family="bcnPower")
xdata$bcn.freq_ff=((0.5*(xdata$freq_ff+sqrt(xdata$freq_ff^2+xx$gamma^2)))^xx$lambda-
1)/xx$lambda
xdata$z.bcn.freq_ff=as.vector(scale(xdata$bcn.freq_ff))
xdata$z.perc_m2_ef=as.vector(scale(xdata$perc_m2_ef))

#MODEL 1
xdata$prop_males=xdata$male/(xdata$male+xdata$female)##martin, the way you determined
prop_males (xdata$male/sum(xdata$male, xdata$female)) didn't really do the right thing
xdata$z.prop_males=as.vector(scale(xdata$prop_males))
xdata$prop.male.submissions=cbind(xdata$fm_dom, xdata$mf_dom)
xdata$olre=as.factor(1:nrow(xdata))
full.prop_males.bin=glmer(prop.male.submissions~z.prop_males+(1+z.prop_males||com) +
(1|olre), data=xdata, family=binomial, control=glmerControl(optimizer="bobyqa",
optCtrl=list(maxfun=10000)))
overdisp.test(full.prop_males.bin)
null.prop_males.bin=glmer(prop.male.submissions~1+(1+z.prop_males||com) + (1|olre),
data=xdata, family=binomial, control=glmerControl(optimizer="bobyqa",
optCtrl=list(maxfun=10000)))
as.data.frame(anova(null.prop_males.bin, full.prop_males.bin, test="Chisq"))
round(summary(full.prop_males.bin)$coefficients, 3)
tests.full.prop_males.bin=as.data.frame(drop1(full.prop_males.bin, test="Chisq"))

#MODEL 2A (model with perc_m2_ef as random slope and prop fem win underlying
dominance)

xdata$prop.f.win=cbind(xdata$fm_win, xdata$mf_win)
  xdata$olre=as.factor(1:nrow(xdata))
full.bcn.freq_ff_ef.bin=glmer(prop.f.win~z.bcn.freq_ff+z.perc_m2_ef+(1+z.perc_m2_ef||group)
+ (1|olre), data=xdata, family=binomial, control=glmerControl(optimizer="bobyqa",
optCtrl=list(maxfun=10000)))
```

```
overdisp.test(full.bcn.freq_ff_ef.bin)
null.bcn.freq_ff_ef.bin=glmer(prop.f.win~1+(1+z.perc_m2_ef||group) + (1|olre), data=xdata,
family=binomial, control=glmerControl(optimizer="bobyqa", optCtrl=list(maxfun=10000)))
as.data.frame(anova(null.bcn.freq_ff_ef.bin, full.bcn.freq_ff_ef.bin, test="Chisq"))
round(summary(full.bcn.freq_ff_ef.bin)$coefficients, 3)
tests.full.bcn.freq_ff_ef.bin=as.data.frame(drop1(full.bcn.freq_ff_ef.bin, test="Chisq"))

#MODEL 2B (model with freq_ff as random slope and prop fem win underlying dominance)
full.bcn.freq_ff_ff.bin=glmer(prop.f.win~z.bcn.freq_ff+z.perc_m2_ef+(1+z.bcn.freq_ff||group) +
(1|olre), data=xdata, family=binomial, control=glmerControl(optimizer="bobyqa",
optCtrl=list(maxfun=10000)))
overdisp.test(full.bcn.freq_ff_ff.bin)
null.bcn.freq_ff_ff.bin=glmer(prop.f.win~1+(1+z.bcn.freq_ff||group) + (1|olre), data=xdata,
family=binomial, control=glmerControl(optimizer="bobyqa", optCtrl=list(maxfun=10000)))
as.data.frame(anova(null.bcn.freq_ff_ff.bin, full.bcn.freq_ff_ff.bin, test="Chisq"))
round(summary(full.bcn.freq_ff_ff.bin)$coefficients, 3)

#MODEL 3A (model with perc_m2_ef as random slope and DS underlying dominance)
beta.tr<-function(x){
   if(any(is.na(x))){warning("x comprises NAs")}
   return((x*(length(x) - 1) + 0.5)/length(x))
 }
 xdata$tr.female_DS_dom=beta.tr(xdata$female_DS_dom)
full.perc_m2_ef.prop=glmmTMB(tr.female_DS_dom~z.bcn.freq_ff+z.perc_m2_ef+(1+z.perc_m
2_ef||group), data=xdata, family=beta_family)
null.perc_m2_ef.prop=glmmTMB(tr.female_DS_dom~1+(1+z.perc_m2_ef||group), data=xdata,
family=beta_family)
overdisp.test(full.perc_m2_ef.prop)
as.data.frame(anova(null.perc_m2_ef.prop, full.perc_m2_ef.prop, test="Chisq"))
round(summary(full.perc_m2_ef.prop)$coefficients$cond, 3)

#MODEL 3B (model with freq_ff as random slope and DS underlying dominance)
full.freq_ff.prop=glmmTMB(tr.female_DS_dom~z.bcn.freq_ff+z.perc_m2_ef+(1+z.bcn.freq_ff||g
roup), data=xdata, family=beta_family)
null.freq_ff.prop=glmmTMB(tr.female_DS_dom~1+(1+z.bcn.freq_ff||group), data=xdata,
family=beta_family)
as.data.frame(anova(null.freq_ff.prop, full.freq_ff.prop, test="Chisq"))
round(summary(full.freq_ff.prop)$coefficients$cond, 3)
overdisp.test(full.freq_ff.prop)
```
